# Implant-based multi-parameter telemonitoring of patients with heart failure and a defibrillator with vs. without cardiac resynchronization therapy option: a subanalysis of the IN-TIME trial

**DOI:** 10.1007/s00392-019-01447-5

**Published:** 2019-03-14

**Authors:** Johann Christoph Geller, Thorsten Lewalter, Niels Eske Bruun, Milos Taborsky, Frank Bode, Jens Cosedis Nielsen, Christoph Stellbrink, Steffen Schön, Holger Mühling, Hanno Oswald, Sebastian Reif, Stefan Kääb, Peter Illes, Jochen Proff, Nikolaos Dagres, Gerhard Hindricks, For the IN-TIME Study Group, Johann Christoph Geller, Thorsten Lewalter, Niels Eske Bruun, Milos Taborsky, Frank Bode, Jens Cosedis Nielsen, Christoph Stellbrink, Steffen Schön, Holger Mühling, Hanno Oswald, Sebastian Reif, Stefan Kääb, Peter Illes, Jochen Proff, Nikolaos Dagres, Gerhard Hindricks

**Affiliations:** 1 Arrhythmia and Invasive Electrophysiology Section, Division of Cardiology, Zentralklinik Bad Berka, Bad Berka, Germany; 2 Otto-von-Guericke University School of Medicine, Magdeburg, Germany; 3 University Hospital Bonn, Bonn, Germany; 4 Present Address: Department of Cardiology, Hospital Munich-Thalkirchen, Peter Osypka Heart Center, Munich, Germany; 5 Department of Cardiology, University Hospital Gentofte, Hellerup, Denmark; 6 Na Homolce, Prague, Czech Republic; 7 Present Address: Department of Internal Medicine I-Cardiology, Faculty of Medicine and Dentistry, Olomouc, Czech Republic; 8 II. Department of Medicine-Cardiology, Angiology and Intensive Care Medicine, Universitätsklinikum Schleswig-Holstein, Lübeck, Germany; 9 Present Address: Department of Cardiology, Sana Kliniken Ostholstein, Klinik Oldenburg, Oldenburg, Germany; 10 Department of Clinical Medicine, Århus University, Åarhus, Denmark; 11 Clinic for Cardiology and Internistic Intensive Care Medicine, Bielefeld Clinical Center, Bielefeld, Germany; 12 Division of Cardiology, Vascular Medicine, Pneumology and Intensive Care Medicine, HELIOS Klinikum Pirna GmbH, Pirna, Germany; 13 Medical Center for Cardiology, Munich, Germany; 14 Clinic for Cardiology and Angiology, Hannover Medical School, Hannover, Germany; 15 Department of Cardiology and Internistic Intensive Care Medicine, Munich State Hospital Bogenhausen, Munich, Germany; 16 Department of Medicine I, Cardiology, Klinikum Großhadern, Munich, Germany; 17 Sydney Adventist Hospital, Sydney, Australia; 18 Center for Clinical Research, Biotronik SE & Co. KG, Berlin, Germany; 19 Department of Electrophysiology, University of Leipzig, Heart Center, Leipzig, Germany

**Keywords:** Telemonitoring of patients with heart failure, Remote monitoring of implantable cardioverter-defibrillators, Remote monitoring of cardiac resynchronization therapy defibrillators

## Abstract

**Aims:**

In the IN-TIME trial, automatic daily implant-based multiparameter telemonitoring significantly improved clinical outcomes in patients with chronic systolic heart failure and implantable cardioverter-defibrillator (ICD) or cardiac resynchronization therapy defibrillator (CRT-D). We compared IN-TIME results for ICD and CRT-D subgroups.

**Methods:**

Patients with LVEF ≤ 35%, NYHA class II/III, optimized drug treatment, no permanent atrial fibrillation, and a dual-chamber ICD (*n* = 274) or CRT-D (*n* = 390) were randomized 1:1 to telemonitoring or no telemonitoring for 12 months. Primary outcome measure was a composite clinical score, classified as worsened if the patient died or had heart failure-related hospitalization, worse NYHA class, or a worse self-reported overall condition.

**Results:**

The prevalence of worsened score at study end was higher in CRT-D than ICD patients (26.4% vs. 18.2%; *P* = 0.014), as was mortality (7.4% vs. 4.1%; *P* = 0.069). With telemonitoring, odds ratios (OR) for worsened score and hazard ratios (HR) for mortality were similar in the ICD [OR = 0.55 (*P* = 0.058), HR = 0.39 (*P* = 0.17)] and CRT-D [OR = 0.68 (*P* = 0.10), HR = 0.35 (*P* = 0.018)] subgroups (insignificant interaction, *P* = 0.58–0.91).

**Conclusion:**

Daily multiparameter telemonitoring has a potential to reduce clinical endpoints in patients with chronic systolic heart failure both in ICD and CRT-D subgroups. The absolute benefit seems to be higher in higher-risk populations with worse prognosis.

**Electronic supplementary material:**

The online version of this article (10.1007/s00392-019-01447-5) contains supplementary material, which is available to authorized users.

## Introduction

Heart failure is associated with high morbidity and poor prognosis [1]. Implantable cardioverter-defibrillators (ICDs) are frequently used in this population to prevent sudden arrhythmic death [2, 3]. Hospitalizations and deaths caused by heart failure may be preceded by changes in clinical parameters such as ventricular tachyarrhythmia, onset of atrial fibrillation, or lung fluid accumulation [4, 5]. These and other potential precursors of heart failure events can be monitored remotely by modern ICDs [4–13].

The IN-TIME trial (INfluence of home moniToring on mortality and morbidity in heart failure patients with IMpaired lEft ventricular function) recently showed that automatic, implant-based multiparameter telemonitoring improves clinical outcome in heart failure patients with ICDs or cardiac resynchronization therapy defibrillators (CRT-Ds) [9]. It is currently unknown whether the benefit of remote monitoring is similar in patients with ICDs and those with implanted CRT-Ds (i.e., whether CRT-D patients have more benefit from monitoring). Therefore, the present IN-TIME subanalysis explores differences between ICD and CRT-D patients in the endpoint rate and in the benefit of telemonitoring.

## Methods

The prospective, multicenter, randomized, controlled IN-TIME trial enrolled patients with chronic heart failure (≥ 3 months) and New York Heart Association (NYHA) functional class II or III symptoms, a left ventricular ejection fraction (LVEF) ≤ 35%, optimized drug therapy, no permanent atrial fibrillation, and a recently implanted Lumax^®^ dual-chamber ICD or Lumax^®^ CRT-D capable of automatic daily multiparameter telemonitoring (Home Monitoring; Biotronik SE & Co. KG, Berlin, Germany) [9]. The decision to implant an ICD or a CRT-D was at the investigator’s discretion based on disease condition.

At 1 month after implantation, patients were randomly assigned on a 1:1 basis to receive telemonitoring in addition to standard care or to standard care alone without telemonitoring for 12 months [9]. In the telemonitoring group, transmitted data were reviewed by the study investigators and by a central monitoring unit located at the Heart Center Leipzig, Germany. The role of this unit was to ensure the awareness of investigational sites for pre-defined medical events such as ventricular and atrial tachyarrhythmia episodes, low percentage of biventricular pacing, increase in the frequency of ventricular extrasystoles, decreased patient activity, and abnormal intracardiac electrograms transmitted in conjunction with detected arrhythmias. The clinical response to telemonitoring observations remained at the discretion of the investigators. They reported whether an additional clinical follow-up was scheduled and whether a visit to the general practitioner was recommended [9].

In the control group, no study participant had access to telemonitoring data until study completion. In both randomization groups, patients were treated according to European guidelines, and investigators decided on the need for follow-up visits, except for the mandatory 12-month visit after randomization. At each follow-up visit, NYHA classification was re-assessed, and patients graded their overall condition as unchanged or slightly, moderately, or markedly worsened, or improved since randomization (global self-assessment) [9].

### Outcome measures

The primary outcome measure was a worsened composite clinical score at 12 months in the intention-to-treat population [9]. The score was classified as worsened if the patient died, had an overnight admission to hospital associated with worsening heart failure, had a worse NYHA functional class, or had a moderately to markedly worse self-reported overall condition compared with that at randomization [14]. An endpoint committee (see Online Resource 1), blinded to treatment allocation, judged endpoints and verified the composite clinical score for each patient. The clinically relevant secondary outcome measures were all-cause mortality and overnight admission to hospital associated with worsening heart failure [9].

### Statistical methods

The primary outcome measure was evaluated using odds ratios and a logistic regression model. Time-to-event data were analyzed by the Kaplan–Meier method and compared by the Cox regression model. Continuous data were non-normally distributed and hence compared with the Mann–Whitney–Wilcoxon rank sum test. Categorical data were compared by the exact Pearson’s chi-squared test. The present subanalysis was not pre-specified.

A two-sided *P* value < 0.05 was considered statistically significant. In multiple comparisons of baseline characteristics for ICD vs. CRT-D patients, the threshold for statistical significance was adjusted by the Holm–Bonferroni method. The analyses were conducted with the IBM SPSS 22 for Windows statistical software (IBM Corporation, Armonk, New York, USA). *P* values are presented with two significant digits and up to three decimal places.

## Results

### Clinical outcomes for ICD versus CRT-D patients

Among 664 patients randomized at 36 investigational sites in seven countries (see Appendix), 274 patients received a dual-chamber ICD (41.3%) and 390 CRT-D (58.7%). Baseline patient characteristics are shown in Table 1. CRT-D patients were significantly older (median age 68 vs. 65 years) and sicker than ICD recipients, with a lower LVEF (median 25% vs. 28%; *P* < 0.001), higher prevalence of NYHA class III symptoms (73.8% vs. 33.2%; *P* < 0.001), and a longer intrinsic QRS duration (median 150 vs. 110 ms; *P* < 0.001). Ischemic heart disease was, however, more prevalent in ICD patients (79.9% vs. 61.3%; *P* < 0.001).

**Table 1:** Characteristics of patients at enrolment

Characteristics	ICD (*n* = 274)	CRT-D (*n* = 390)	*P* value^a^ ICD vs. CRT-D
Age, years	65 [58–70]	68 [62–74]	*< 0.001*
Male gender	233 (85.0%)	303 (77.7%)	0.021
Body mass index	27.5 [24.7–31.1]	27.5 [24.6–30.5]	0.75
LVEF^b^, %	28.0 [24.5–30.0]	25.0 [20.0–30.0]	*< 0.001*
NYHA^c^			*< 0.001*
Class II	183 (66.8%)	102 (26.2%)	n.a
Class III	91 (33.2%)	287 (73.8%)	n.a
Intrinsic QRS duration, ms	110 [110–124]	150 [130–165]	*< 0.001*
Resting heart rate, beats/min	70 [60–78]	70 [60–80]	0.27
Indication for defibrillator			
Primary prevention	204 (74.5%)	321 (82.3%)	0.016
Secondary prevention	70 (25.5%)	69 (17.7%)	n.a
Medical history			
Coronary artery disease	219 (79.9%)	239 (61.3%)	*< 0.001*
Stroke	19 (6.9%)	42 (10.8%)	0.10
Transient ischemic attack	2 (0.7%)	11 (2.8%)	0.085
Hypertension	187 (68.2%)	276 (70.8%)	0.49
Atrial fibrillation	67 (24.5%)	101 (25.9%)	0.72
Paroxysmal	43 (15.8%)	69 (17.7%)	n.a.
Persistent	23 (8.4%)	30 (7.7%)	n.a.
COPD	39 (14.2%)	55 (14.1%)	1.0
Diabetes mellitus	102 (37.2%)	164 (42.1%)	0.23
Renal insufficiency	67 (24.5%)	132 (33.8%)	0.010
Medication			
Diuretic	252 (92.0%)	368 (94.4%)	0.27
Spironolactone	138 (50.4%)	219 (56.2%)	0.16
ACE inhibitor or ARB	251 (91.6%)	342 (87.7%)	0.13
Beta blocker	249 (90.9%)	359 (92.1%)	0.67
Any antiarrhythmic	41 (15.0%)	65 (16.7%)	0.59
Anticoagulant	80 (29.2%)	123 (31.5%)	0.55

Data are presented as median [interquartile range] and *n* (%) of patients. For mean values and additional patient characteristics at enrolment, see Online Resource 2

*ACE* angiotensin-converting enzyme, *ARB* angiotensin receptor blocker, *COPD* chronic obstructive pulmonary disease, *CRT-D* cardiac resynchronization therapy defibrillator, *ICD* implantable cardioverter-defibrillator, *LVEF* left ventricular ejection fraction, *n.a*. not applicable, *NYHA* New York Heart Association, *SD* standard deviation

^a^Because multiple parameters were tested, the threshold of significance was determined using the Holm–Bonferroni method, applied separately for the medication block (15 parameters) and for the other 18 parameters (full list of parameters in Online Resource 2). Significant *P* values according to this method are italicized (all were ≤ 0.002). Variables with the *P* value “n.a.” were not included in Holm–Bonferroni method because they were not sufficiently independent

^b^Determined within 3 months before enrollment

^c^Unknown in one CRT-D patient

Median length of follow-up after randomization was 350 days in the ICD group (mean ± standard deviation, 334 ± 80) and 353 days (328 ± 88) in the CRT-D group. The prevalence of worsened composite clinical score at study end was higher in CRT-D than ICD patients (26.4% vs. 18.2%; *P* = 0.014). Table 2 summarizes the underlying reasons for worsened score. The prevalence of improved composite clinical score was also higher in CRT-D patients (35.9% vs. 27.7%; *P* = 0.027).

**Table 2:** Individual components of the composite clinical score

Composite clinical score	ICD patients			CRT-D patients		
	Telemon. (*n* = 143)	Control (*n* = 131)	Total (*n* = 274)	Telemon. (*n* = 190)	Control (*n* = 200)	Total (*n* = 390)
Worsened	20 (14.0%)	30 (22.9%)	50 (18.2%)	43 (22.6%)	60 (30.0%)	103 (26.4%)
Death	3 (2.1%)	7 (5.3%)	10 (3.6%)	7 (3.7%)	20 (10.0%)	27 (6.9%)
Overnight admission to hospital for WHF	9 (6.3%)^a^	7 (5.3%)^a^	16 (5.8%)^a^	14 (7.4%)^a^	20 (10.0%)^a^	34 (8.7%)^a^
Worse NYHA class	7 (4.9%)^a^	16 (12.2%)^a,b^	23 (8.4%)^a^	16 (8.4%)^a^	16 (8.0%)^a^	32 (8.2%)^a^
Worse global self-assessment	1 (0.7%)^a^	1 (0.8%)^a,b^	2 (0.7%)^a^	6 (3.1%)^a^	4 (2.0%)^a^	10 (2.6%)^a^
Improved^c^	42 (29.4%)	34 (26.0%)	76 (27.7%)	69 (36.3%)	71 (35.5%)	140 (35.9%)
Unchanged	81 (56.6%)	67 (51.1%)	148 (54.0%)	78 (41.1%)	69 (34.5%)	147 (37.7%)

Data are *n* (%)

*CRT-D* cardiac resynchronization therapy defibrillator, *ICD* implantable cardioverter-defibrillator, *NYHA* New York Heart Association, *telemon*. telemonitoring, *WHF* worsening heart failure

^a^Patients are included only once, in the topmost subcategory

^b^One patient had worsened both NYHA class and global self-assessment

^c^Improved NYHA class or moderately to markedly improved self-assessed condition in those who did not die or have WHF hospitalization

The 1-year Kaplan–Meier estimate for the composite of all-cause mortality and heart failure-related hospitalization was significantly higher in the CRT-D than ICD group (16.3% vs. 9.3%; *P* = 0.008), with a similar trend for mortality alone (7.4% vs. 4.1%; *P* = 0.069) and heart failure hospitalization alone (11.9% vs. 7.5%; *P* = 0.046).

### Clinical outcomes for telemonitoring versus no telemonitoring

**Fig. 1:**
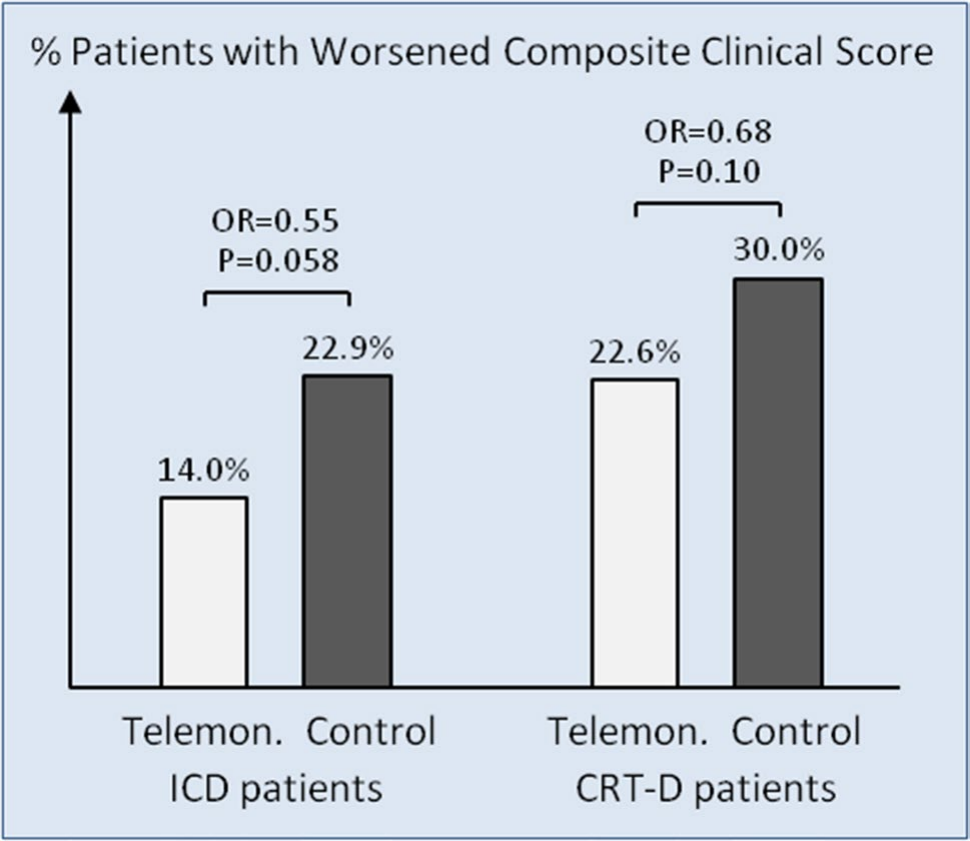
Percentage of patients with worsened composite clinical score. For the number of patients in each group, see Table 2. *CRT-D* cardiac resynchronization therapy defibrillator, *ICD* implantable cardioverter-defibrillator, *OR* odds ratio, *telemon*. telemonitoring

The odds ratio for a worsened composite clinical score with telemonitoring vs. no telemonitoring was similar for ICD (0.55; *P* = 0.058) and CRT-D (0.68; *P* = 0.10) patients (Fig. 1), without a significant statistical interaction (*P* = 0.58).

**Fig. 2:**
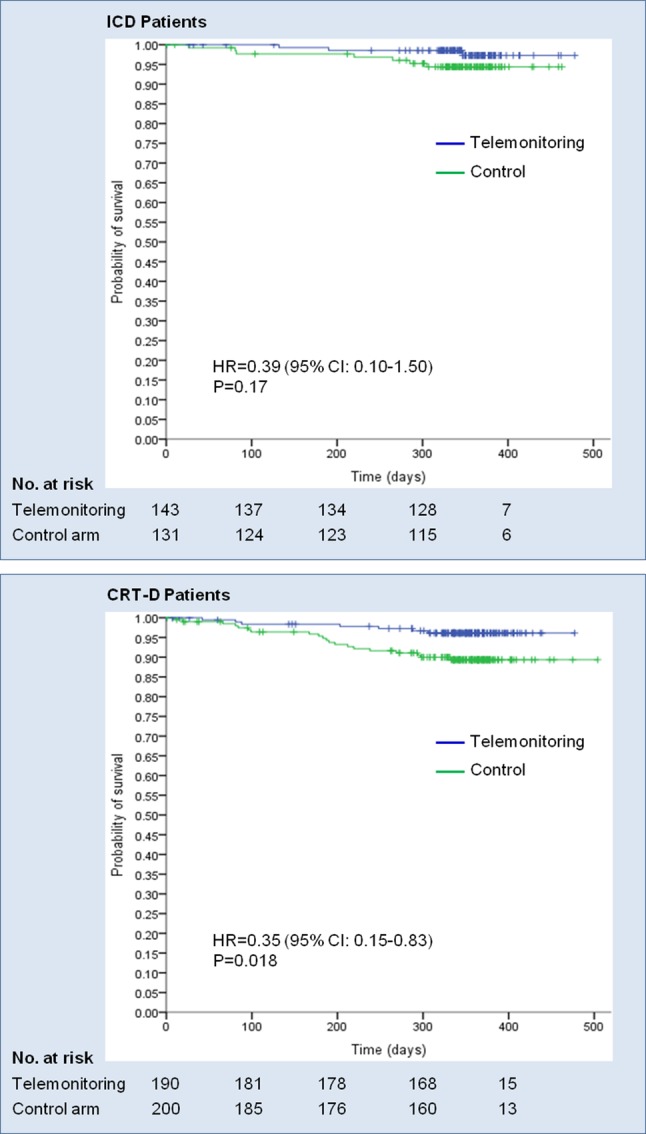
Kaplan–Meier curves of patient survival. *CI* confidence interval, *CRT-D* cardiac resynchronization therapy defibrillator, *ICD* implantable cardioverter-defibrillator

The 1-year Kaplan–Meier estimates of mortality for telemonitoring vs. usual care were 2.7% vs. 5.6% (ICD) and 3.9% vs. 10.7% (CRT-D). The hazard ratio was very similar for ICD (0.39; *P* = 0.17) and CRT-D (0.35; *P* = 0.018) patients (Fig. 2), without significant interaction (*P* = 0.91).

The total number of overnight hospital admissions for worsening heart failure in the telemonitoring vs. control arm was 14 vs. 13 (ICD) and 30 vs. 34 (CRT-D). The number of affected patients was 10 vs. 8 (7.0% vs. 6.1%; *P* = 0.81; ICD) and 17 vs. 26 (8.9% vs. 13.0%; *P* = 0.26; CRT-D), the median length of hospital stay was 9.0 vs. 7.0 days (ICD; *P* = 0.38) and 7.0 vs. 7.5 days (CRT-D; *P* = 0.43), respectively. In contrast to Table 2, these numbers also include hospitalized patients who died during the study.

### Patient contacts in response to telemonitoring data

Telemonitoring data and the related patient contacts are summarized per device type in Table 3. The central monitoring unit forwarded telemonitoring observations to the investigational sites for the great majority of patients from the telemonitoring arm irrespective of device type. Thus, in a total of 117 ICD patients (82% of telemonitored ICD patients), 471 observations were forwarded during 132 cumulative patient-years of follow-up, corresponding to 3.6 observations per patient-year. In the same time, in 163 CRT-D patients (86% of telemonitored CRT-D patients), 754 observations were forwarded during 175 cumulative patient-years, corresponding to 4.3 observations per patient-year. The moderately higher number of forwarded observations in the CRT-D subgroup (4.3 vs. 3.6 per patient-year) is attributable to the CRT-D-specific alert for low percentage of biventricular pacing (“CRT < 80% over 48 h”, Table 3).

**Table 3:** Telemonitoring observations and reactions^a^

	Observation sent to IS	Patient contact by IS	Further action by IS^b^
ICD patients: 143 on telemonitoring			
Ventricular tachyarrhythmia or shock^c^	15 (22)	11 (19)	6 (11)
Atrial tachyarrhythmia^d^	31 (58)	25 (34)	10 (13)
CRT < 80% over 48 h	n.a.	n.a.	n.a.
VES frequency > 110 per hour or increasing trend over 7 days	20 (23)	13 (15)	2 (2)
Decreasing trend of patient activity over 7 days	0 (0)	0 (0)	0 (0)
Abnormal IEGM, or sensing safety notification^d^	11 (22)	6 (7)	5 (5)
Pacing or impedance safety notification^e^	4 (5)	3 (3)	1 (1)
Gap in data transmission of > 3 days	101 (339)	70 (147)	1 (1)
Total	117 (471)	97 (226)	23 (33)
Mean per patient-year	3.6	1.7	0.25
CRT-D patients: 190 on telemonitoring			
Ventricular tachyarrhythmia or shock^c^	27 (34)	14 (19)	9 (11)
Atrial tachyarrhythmia^d^	34 (51)	28 (36)	8 (11)
CRT < 80% over 48 h	35 (91)	28 (63)	15 (26)
VES frequency > 110 per hour or increasing trend over 7 days	26 (31)	21 (24)	5 (5)
Decreasing trend of patient activity over 7 days	1 (1)	1 (1)	0 (0)
Abnormal IEGM, or sensing safety notification^e^	24 (31)	14 (18)	9 (10)
Pacing or impedance safety notification^f^	22 (38)	10 (11)	4 (4)
Gap in data transmission of > 3 days	140 (480)	104 (254)	3 (3)
Total	163 (754)	141 (415)	40 (66)
Mean per patient-year	4.3	2.4	0.39
Mean per patient-year excluding “CRT < 80% over 48 h”	3.8	1.9	0.23

Data are number of patients (number of events) unless stated otherwise

Observations were forwarded by the central monitoring unit to investigational sites

*CRT* percentage of biventricular pacing, *CRT-D* cardiac resynchronization therapy defibrillator, *ICD* implantable cardioverter-defibrillator, *IEGM* intracardiac electrogram, *IS* investigational site, *n.a*. not applicable, *VES* ventricular extrasystole

^a^Differences between ICD and CRT-D patients were not tested for statistical significance because of multiplicity issues and the lack of pre-defined hypotheses with margins of relevance

^b^A scheduled clinical follow-up or a suggested patient visit to the general practitioner

^c^Could include inappropriate detections

^d^The first onset of atrial fibrillation for > 30 s, a long atrial arrhythmia episode (≥ 6 h) with high ventricular rate (> 120 beats per minute), or high atrial arrhythmia daily burden (≥ 50%) on 7 consecutive days

^e^Abnormal IEGM: T-wave oversensing, far-field atrial sensing of ventricular activity, or other suspected sensing problem. Sensing safety notification: low sensing amplitude or insufficient safety margin on any lead

^f^Pacing safety notification: low safety margin for stimulation on right or left ventricular lead. Impedance safety notification: out-of-range impedance of any lead

In response to the telemonitoring data, investigators contacted 97 ICD patients on 226 occasions (1.7 contacts per patient-year, involving 68% of telemonitored ICD patients) and 141 CRT-D patients on 415 occasions (2.4 contacts per patient-year, involving 74% of telemonitored CRT-D patients). Details are given in Table 3.

As a result, 23 ICD patients were invited to 33 additional follow-up visits to a specialized center for device follow-up or to the general practitioner (corresponding to 0.25 extra visits per patient-year, needed in 16% of telemonitored ICD patients), and 40 CRT-D patients were invited to 66 extra visits (0.39 extra visits per patient-year, needed in 21% of telemonitored CRT-D patients).

### Percentage of ventricular pacing

Percentage of right ventricular pacing was evaluated in ICD patients, since high prevalence of pacing may worsen clinical outcomes [15–17]. The Home Monitoring Service Center provided averaged data per patient over the randomized period. The median value of 0.6% right ventricular pacing (interquartile range 0–4.3%; mean ± standard deviation, 8.8 ± 21.0%; similar for telemonitoring vs. no telemonitoring group) should not have influenced clinical outcomes. In contrast, the percentage of biventricular pacing in CRT-D patients should be as high as possible [18]. The median value of 98.6% (interquartile range 96.4–99.6%; mean ± standard deviation, 96.1 ± 8.6%) shows that this goal was indeed achieved.

## Discussion

### Main findings

In patients with chronic systolic heart failure (LVEF ≤ 35%), (1) worsened composite clinical score after 1 year (primary outcome) occurred more frequently in CRT-D than ICD patients (26.4% vs. 18.2%), (2) improved score after 1 year also occurred more frequently in CRT-D patients (35.9% vs. 27.7%), and (3) the effect of telemonitoring did not differ between ICD and CRT-D patients in terms of odds ratios for worsened score (range 0.55–0.68) and hazard ratios for mortality (0.35–0.39).

### Composite clinical score (primary outcome)

The composite clinical score (“Packer score”) is relatively new and was designed specifically for patients with heart failure [14]. Before IN-TIME, three large trials (REVERSE [19], PROSPECT [20], PEGASUS CRT [21]) used this score in patients with cardiac resynchronization therapy (CRT) or CRT-D devices (all without telemonitoring), but only PEGASUS CRT patients had similar baseline characteristics (mean age 67 ± 11 years, LVEF 23.5 ± 6.5%, predominantly NYHA III) as the CRT-D patients in IN-TIME. The rate of worsened score in PEGASUS CRT was 25–28% (depending on CRT-D programming) after 10.5 ± 3.5 months [21], comparable to the 30% rate after 10.8 ± 2.9 months in the CRT-D subgroup without telemonitoring in IN-TIME. The other two studies had only 6-month follow-up [20] or dealt with milder heart failure [19], resulting in a 16% rate of worsened score [19, 20]. We are not aware of a large trial other than IN-TIME reporting this endpoint in CRT(-D) patients using telemonitoring, or in ICD patients.

The present subanalysis revealed a significantly higher rate of worsened score in CRT-D than in ICD patients (30.0% vs. 22.9% without telemonitoring and 22.6% vs. 14.0% with telemonitoring), which may be attributed to more advanced heart failure (lower LVEF, higher NYHA class) and higher age of CRT-D patients. Although telemonitoring was associated with a consistent numerical reduction of worsened score in both ICD and CRT-D subgroups (odds ratio 0.55–0.68), the division of patients into subgroups reduced statistical power, and the *P* values narrowly missed significance (*P* = 0.058–0.10). In pooled data, the odds ratio was 0.63, *P* = 0.013 [9].

The rate of improved score at 1 year (better NYHA class or improved global self-assessment) was also significantly higher in CRT-D than ICD patients (35.9% vs. 27.7%), possibly due to the benefit of chronic cardiac resynchronization in patients with left ventricular dyssynchrony [1]. There was no effect of telemonitoring on score improvement.

### Mortality

IN-TIME is the only randomized controlled trial showing a reduction in all-cause mortality with implant-based telemonitoring vs. no telemonitoring (the 1-year Kaplan–Meier estimate, 3.4% vs. 8.7%; hazard ratio 0.36, *P* = 0.004) [9]. The present subanalysis indicates no major difference between device types (hazard ratio 0.35–0.39, *P* = 0.018–0.17). In line with this, large-scale US-based non-randomized registries, each enrolling > 140,000 ICD and CRT-D patients (ALTITUDE [8] and Varma et al. [22]), observed significantly fewer deaths with telemonitoring in both CRT-D and ICD subgroups (hazard ratio 0.45–0.67, *P* < 0.001), although bias inherent to non-randomized study designs cannot be excluded in these registries. In contrast, neutral mortality results were reported in a number of randomized trials of up to 2000 patients, using various telemonitoring systems and settings [12, 13, 23–25]. Thus, the clinical benefit may depend on details of the used technology, patient selection, and clinical reaction to telemonitoring data [26].

In the CRT-D subgroup in IN-TIME, the 1-year estimate of all-cause mortality irrespective of the randomization group was 7.4%. This is higher than the mortality of CRT-D patients in two important reference studies, PEGASUS CRT (4.1–6.4% at 1 year, derived from the 3.6–5.6% rate at 10.5 ± 3.5 months) [21] and MORE-CARE (the 2-year estimate 10.3%; no 1-year data) [12]. While PEGASUS CRT and IN-TIME patients had similar baseline characteristics (see above), patients enrolled in MORE-CARE had slightly better LVEF (27.4 ± 6.0%) than CRT-D patients in IN-TIME (25.0 ± 6.5%) [12]. MORE-CARE is the only large, randomized, outcome trial of implant-based telemonitoring in patients with CRT-D devices; the other trials enrolled either ICD patients (TRUST [27], ECOST [28]) or both ICD and CRT-D patients without reporting CRT-D results separately (CONNECT [29], OptiLink HF [23], REM-HF [13]). Furthermore, the large ALTITUDE [8] and Varma et al. [22] registries, and another non-randomized telemonitoring study of 570 ICD and 417 CRT-D patients (De Simone et al. [30]), reported 12% [8], 6.6% [22], and 6.5% [30] mortality rates among CRT-D patients at 1 year. Altogether, mortality of CRT-D patients in IN-TIME was lower than in ALTITUDE, but higher than in all other studies included in this comparison.

A similar literature analysis revealed that the 1-year estimate of mortality in ICD patients in IN-TIME (4.1%) was lower than in ALTITUDE (8%) [9] and De Simone et al. (6.0%) [30], but similar to Varma et al. (4.5%) [22], TRUST (4.1%) [27], and ECOST (9.7% at 2 years) [28]. Moreover, all studies including both device types reported a higher mortality in CRT-D than ICD patients (12% vs. 8% [8], 6.6% vs. 4.5% [22], 6.5% vs. 6.0% [30], and 7.4% vs. 4.1% in IN-TIME). This trend is in line with the higher rate of worsened Packer score in CRT-D vs. ICD patients in IN-TIME, discussed above.

### Heart failure-related hospitalizations

Telemonitoring did not significantly influence hospital admissions for worsening heart failure in IN-TIME [9]. The present subanalysis indicates a trend toward fewer admissions in CRT-D but not ICD patients under telemonitoring. While heart failure hospitalizations require a careful, blinded adjudication and are rarely reported as a separate category in studies of remote ICD or CRT-D monitoring, a meta-analysis of these trials showed no reduction in the overnight hospital admissions for all cardiac causes with telemonitoring (relative risk 0.96, *P* = 0.60) [25].

It is noteworthy that another telemonitoring system, a stand-alone implantable monitor of pulmonary artery pressure (CardioMems Heart Failure Sensor; CardioMems, Atlanta, Georgia) can reduce the risk of recurrent hospitalizations in symptomatic (NYHA class III), previously hospitalized heart failure patients irrespective of the LVEF [1, 31–33]. Thus, in the CHAMPION trial, a 33% hospitalization reduction was reported in patients randomized to pre-specified treatment guided by daily pulmonary artery pressure measurements vs. standard care (*P* < 0.0001) [31, 32]; however, mortality was not reduced significantly.

### Benefit of telemonitoring in CRT-D versus ICD patients

In a recent meta-analysis of IN-TIME [9], ECOST [28], and TRUST [27] trials using the same telemonitoring system (Biotronik Home Monitoring), Hindricks et al. [34] confirmed the IN-TIME findings on all-cause mortality and concluded that the benefit of daily automated Home Monitoring over standard in-office follow-up is largely driven by the prevention of worsening heart failure events (deaths in IN-TIME, hospitalizations for heart failure in ECOST). Accordingly, patients with more advanced heart failure may gain a greater clinical benefit.

In IN-TIME, both CRT-D and ICD patients were at risk of heart failure events, but the risk was greater in CRT-D recipients who in the end had a higher rate of worsened Packer score (26.4% vs. 18.2%), mortality (7.4% vs. 4.1%), the composite of mortality and heart failure hospitalization (16.3% vs. 9.3%), and heart failure hospitalization (11.9% vs. 7.5%) than ICD recipients. This was also in line with the comparatively worse baseline characteristics of CRT-D patients (older, more advanced heart failure). The observed greater absolute benefit of telemonitoring in the CRT-D subgroup (e.g., mortality reduction by an absolute 6.8% vs. 2.9% in ICD patients) is in agreement with (1) more telemonitoring alerts per patient-year (+ 19%), (2) more triggered contacts to patients (+ 41%), and (3) more additional follow-up visits (+ 56%) than in the ICD subgroup. If more therapy modifications are triggered by telemonitoring per patient-year, it is plausible that also more endpoints can be prevented.

On the other hand, the odds ratios for the reduction in primary outcome by remote monitoring were similar in ICD and CRT-D patients (0.55–0.68), as were hazard ratios for the reduction in mortality (0.35–0.39). While this comparison across device types was neither pre-defined nor statistically powered, it is reassuring that there was not even a weak trend toward a larger relative effect in one device subgroup. In retrospect, this justifies the study design to include ICD patients with and without CRT. It was initially unclear whether poor status of CRT-D patients would offset telemonitoring benefit compared to ICD patients or whether monitoring of biventricular pacing percentage and transmission of more information of relevance would add clinical benefit.

### General discussion

As analyzed above, mortality of CRT-D patients in IN-TIME was higher than in the majority of similar studies, whereas the mortality of ICD patients was comparable. Taken together, the entire IN-TIME population, especially the group without telemonitoring, had a higher incidence of death (8 over 100 patient-years) than the average value (5 over 100 patient-years) in nine randomized implant-based telemonitoring studies included in Table 2 of the meta-analysis by Klersy et al. [25]. The unique findings on telemonitoring benefit in IN-TIME might, therefore, be attributed in part to an overall higher-risk patient cohort enrolled in the study.

This, along with the meta-analysis of Hindricks et al. [34], calls for the implementation of telemonitoring especially in higher-risk patients who have the highest likelihood of gaining a survival benefit. In clinical practice, however, telemedicine seems to be used mainly in patients with better clinical prognosis, probably due to the belief that those who live longer may receive more (i.e., prolonged) benefit from telemonitoring than sicker patients who should be seen in the office more frequently [22, 35].

Debates on the optimal telemonitoring technology (parameters to be monitored, frequency of data transmission) and clinical response system continue [11, 26, 36–38]. Both randomized trials of implant-based telemonitoring with positive outcomes, IN-TIME [9] and CHAMPION [31, 32], were characterized by largely successful daily data transmission and a well-designed response system to device-mediated alerts [26, 39]. The 2016 ESC Guidelines for the Diagnosis and Treatment of Acute and Chronic Heart Failure recommend these two telemonitoring concepts to improve clinical outcomes (IN-TIME approach) or reduce the risk of recurrent heart failure hospitalizations (CardioMems) as class IIb recommendations with the level of evidence B [1].

Recently, another form of remote patient management, using a multicomponent external telemonitoring system (sending daily information of the patient’s weight, blood pressure, heart rhythm, peripheral capillary oxygen saturation, and self-rated health status to a telemedical center) was associated with significantly fewer days lost to unplanned cardiovascular hospital admissions and all-cause death (17.8 vs. 24.2 days per year; *P* = 0.046), and with significantly lower mortality (7.86 vs. 11.34 deaths per 100 person-years of follow-up; *P* = 0.028) than the usual care without remote monitoring in patients with heart failure (NYHA class II or III, hospitalized for heart failure within 12 months before randomization, LVEF ≤ 45% or higher if oral diuretics had been prescribed) and without major depression (TIM-HF2 trial) [40]. The authors conclude that a telemedical center involving physicians and heart failure nurses (preferably for 24 h a day, 7 days a week), and a self-adapting software algorithm with prioritization rules are key elements to enable tailored management of a large number of patients based on individualized risk profiles [40]. The actions taken by the telemedical center staff included changes in medication and hospital admission, if needed, but also educational activities. A holistic approach of interaction between patients, local heart failure caregivers, and a telemedical center enabled intensive and instantaneous outpatient management of heart failure on a daily basis. This experience emphasizes the benefit of optimized organization of care in combination with telemonitoring and intense follow-up with or without implantable device data. By comparison, in the IN-TIME trial, the central monitoring unit informed investigators of protocol-defined events on all working days and investigators contacted patients with a median delay of 1 day and arranged follow-ups, the majority of which took place within 1 week of the event being available [41].

### Study limitations

The three major limitations of the present study are: (1) the limited follow-up period of 12 months; (2) the limited statistical power of post hoc subanalyses in randomized trials; however, clear trends have been observed that may be relevant for clinical practice; (3) therapy changes during follow-up were not collected systematically; hence, we were not able to analyze the role of treatment changes for the clinical benefit in the telemonitoring group.

### Clinical implications

Our results suggest that the intense implant-based multiparameter telemonitoring with daily data transmission has the potential to reduce clinical endpoints in patients with chronic systolic heart failure independent of whether they receive ICD or CRT-D therapy. The absolute benefit seems to be higher in higher risk populations with worse prognosis. These results are especially relevant considering the high numbers of heart failure patients receiving ICDs for prevention of sudden cardiac death.

## Electronic supplementary material

Below is the link to the electronic supplementary material.

Supplementary material 1 (DOC 66 KB)

Supplementary material 2 (DOC 100 KB)
