# Supplementary material for: Implant-based multi-parameter telemonitoring of patients with heart failure and a defibrillator with vs. without cardiac resynchronization therapy option: a subanalysis of the IN-TIME trial
Source: Clin Res Cardiol. 2019 Mar 14;108(10):1117–27. doi: 10.1007/s00392-019-01447-5 (PMC6753058; doi:10.1007/s00392-019-01447-5)
Supplement: Supplementary file 1 — Supplementary material 1 (DOC 66 KB) [file 392_2019_1447_MOESM1_ESM.doc]

**Online Resource 1**

**Clinical Coordinating Investigator:**

Gerhard Hindricks (Leipzig, Germany)

**Other members of the Steering Committee alphabetically:**

Michael Block (Munich, Germany), Josef Kautzner (Prague, Czech Republic), Thorsten Lewalter (Munich, Germany), Stefan Sack (Munich, Germany), Burghard Schuhmacher (Kaiserslautern, Germany), Peter Søgaard (Aalborg, Denmark), Milos Taborsky (Olomouc, Czech Republic). The committee was responsible for developing and monitoring the implementation of study protocol and for ensuring timely publication of results.

**Endpoint Committee:**

Erland Erdmann (Chair; Cologne, Germany), Christoph Hammerstingl (Bonn, Germany), and Christian Kupatt-Jeremias (Munich, Germany) who substituted the deceased Andreas Luchner (Regensburg, Germany). The committee did not participate in the study apart from classifying in a blinded fashion the cause of death (as cardiovascular or non-cardiovascular) and reasons for hospitalization (related or not related to worsening heart failure). The committee also verified in a blinded fashion the composite clinical score for each patient.

**Principal investigators and investigational sites in the alphabetical order of countries and investigators:**

**Australia:** Peter Illes (city: Wahroonga, institution: Sydney Adventist Hospital);

**Austria:** Wolfgang Dichtl (Innsbruck, Uni-klinik für Innere Medizin);

**Czech Republic:** Josef Kautzner (Prague, IKEM); Miloš Taborsky (Prague, Na Homolce);

**Denmark:** Niels Eske Bruun (Hellerup, Gentofte Hospital); Søren Hjortshøj (Aalborg, Aalborg Hospital); Claus Brøckner Nielsen (Aarhus, Uni-hospital, Skejby Sygehus); **Germany:** Steffen Behrens (Berlin, Vivantes Humboldt-Klinikum); Frank Bode (Lübeck, Uni-klinikum Schleswig-Holstein); Johannes Brachmann (Coburg, Klinikum Coburg); Niels Christian Ewertsen (Berlin, Vivantes Klinikum am Urban); Johann-Christoph Geller (Bad Berka, Zentralklinik Bad Berka); Andreas Götte (Paderborn, St. Vincenz Krankenhaus); Ullus Heinrich (Nordhausen, Praxis Dr. Heinrich); Gerhard Hindricks ( Leipzig, Herzzentrum Leipzig); Ellen Hoffmann (München, Herzzentrum München-Bogenhausen); Stefan Kääb (München, Klinikum Großhadern); Steffen Löscher (Leipzig, Klinikum St. Georg); Cartsten Meincke (Berlin, Vivantes Klinikum Neukölln); Holger Mühling (München, Kardiologische Gem.-Praxis Dr. Mühling); Hans-Ruprecht Neuberger (Homburg/Saar, Uni-klinikum des Saarlandes); Hanno Oswald (Hannover, MH Hannnover); Christian Perings (Lünen, St. Marienhospital Lünen); Michael Block (München, Klinik Augustinum); Nico Reinsch (Essen, Westdeutsches Herzzentrum); Stefan Sack (München, Klinikum Schwabing); Anja Schade (Bad Neustadt, Herz- und Gefäß-Klinikum); Alexander Schirdewan (Berlin, Charité – Campus Benjamin Franklin); Rolf Schomburg (Bad Segeberg, Segeberger Kliniken); Steffen Schön (Pirna, Klinikum Pirna); Jörg Schwab (Bonn, Uni-klinikum Bonn); Christoph Stellbrink (Bielefeld, Städtische Kliniken Bielefeld Mitte); Johannes Tebbe (Detmold, Klinikum Lippe-Detmold);

**Israel:** Michael Glikson (Tel-Hashomer, Chaim Sheba Medical Center); Amos Katz (Ashkelon, Barzilai Medical Center);

**Latvia:** Andrejs Erglis (Riga, P. Stradins Clinical University Hospital).
