# Supplementary material for: Implant-based multi-parameter telemonitoring of patients with heart failure and a defibrillator with vs. without cardiac resynchronization therapy option: a subanalysis of the IN-TIME trial
Source: Clin Res Cardiol. 2019 Mar 14;108(10):1117–27. doi: 10.1007/s00392-019-01447-5 (PMC6753058; doi:10.1007/s00392-019-01447-5)
Supplement: Supplementary file 2 — Supplementary material 2 (DOC 100 KB) [file 392_2019_1447_MOESM2_ESM.doc]

**Online Resource 2**

**Table 1. Characteristics of patients at enrolment**

**(full table with all parameters and mean values)**

| **Characteristic** | **ICD**  **(n = 274)** | **CRT-D**  **(n = 390)** | **P-value^a^**  **ICD vs. CRT-D** |
| --- | --- | --- | --- |
|
| Age, years | 65 [58–70] | 68 [62–74] | <0.001 |
| Mean ± SD | 63.5 ± 9.6 | 67.0 ± 9.0 | n.a. |
| Male gender | 233 (85.0%) | 303 (77.7%) | 0.021 |
| Body mass index | 27.5 [24.7–31.1] | 27.5 [24.6–30.5] | 0.75 |
| Mean ± SD | 28.1 ± 4.5 | 28.0 ± 4.6 | n.a. |
| Underweight (index <20) | 6 (2.2%) | 8 (2.1%) | n.a. |
| Obese (index >30) | 88 (32.2%) | 114 (29.6%) | n.a. |
| LVEF^b^, % | 28.0 [24.5–30.0] | 25.0 [20.0–30.0] | <0.001 |
| Mean ± SD | 27.0 ± 6.5 | 25.0 ± 6.5 | n.a. |
| NYHA^c^ |  |  | <0.001 |
| Class II | 183 (66.8%) | 102 (26.2%) | n.a. |
| Class III | 91 (33.2%) | 287 (73.8%) | n.a. |
| Intrinsic QRS duration, ms | 110 [110–124] | 150 [130–165] | <0.001 |
| Mean ± SD | 114 ± 28 | 148 ± 32 | n.a. |
| Resting heart rate, beats/min | 70 [60–78] | 70 [60–80] | 0.27 |
| Mean ± SD | 70 ± 13 | 71 ± 14 | n.a. |
| Indication for defibrillator |  |  |  |
| Primary prevention | 204 (74.5%) | 321 (82.3%) | 0.016 |
| Secondary prevention | 70 (25.5%) | 69 (17.7%) | n.a. |
| SCA, documented VF/VT | 13 (4.7%) | 18 (4.6%) | n.a. |
| SCA, inducible VF/VT | 15 (5.5%) | 11 (2.8%) | n.a. |
| Medical history |  |  |  |
| Coronary artery disease | 219 (79.9%) | 239 (61.3%) | <0.001 |
| Stroke | 19 (6.9%) | 42 (10.8%) | 0.10 |
| Transient ischemic attack | 2 (0.7%) | 11 (2.8%) | 0.085 |
| Hypertension | 187 (68.2%) | 276 (70.8%) | 0.49 |
| Atrial fibrillation | 67 (24.5%) | 101 (25.9%) | 0.72 |
| Paroxysmal | 43 (15.8%) | 69 (17.7%) | n.a. |
| Persistent | 23 (8.4%) | 30 (7.7%) | n.a. |
| COPD | 39 (14.2%) | 55 (14.1%) | 1.0 |
| Diabetes mellitus | 102 (37.2%) | 164 (42.1%) | 0.23 |
| Renal insufficiency | 67 (24.5%) | 132 (33.8%) | 0.010 |
| Hepatic disease | 6 (2.2%) | 9 (2.3%) | 1.0 |
| Mental disorder | 12 (4.4%) | 14 (3.6%) | 0.69 |
| Medication |  |  |  |
| Diuretic | 252 (92.0%) | 368 (94.4%) | 0.27 |
| Spironolactone | 138 (50.4%) | 219 (56.2%) | 0.16 |
| Other diuretic | 221 (80.7%) | 340 (87.2%) | 0.029 |
| ACE inhibitor or ARB | 251 (91.6%) | 342 (87.7%) | 0.13 |
| Beta blocker | 249 (90.9%) | 359 (92.1%) | 0.67 |
| Calcium channel blocker | 27 (9.9%) | 45 (11.5%) | 0.53 |
| Digitalis | 45 (16.4%) | 82 (21.0%) | 0.16 |
| Amiodarone | 22 (8.0%) | 48 (12.3%) | 0.095 |
| Sotalol | 7 (2.6%) | 1 (0.3%) | 0.010 |
| Other antiarrhythmic | 16 (5.9%) | 19 (4.9%) | 0.60 |
| Any antiarrhythmic | 41 (15.0%) | 65 (16.7%) | 0.59 |
| Nitrate | 37 (13.5%) | 45 (11.5%) | 0.47 |
| Lipid lowering agent | 221 (80.7%) | 256 (65.6%) | <0.001 |
| Antiplatelet | 194 (70.8%) | 231 (59.2%) | 0.002 |
| Anticoagulant | 80 (29.2%) | 123 (31.5%) | 0.55 |

Data are presented as median [interquartile range] and mean ± SD, or as n (%) of patients.

^a^Because multiple parameters were tested, the threshold of significance was determined using the Holm-Bonferroni method, applied separately for the medication block (15 parameters) and for the other 18 parameters. Significant P-values according to this method are underlined (all were ≤0.002). Variables with the P-value “n.a.” were not included in Holm-Bonferroni method because they were not sufficiently independent.

^b^Determined within 3 months before enrollment.

^c^Unknown in one CRT-D patient.

Abbreviations: ACE denotes angiotensin-converting enzyme, ARB angiotensin receptor blocker, COPD chronic obstructive pulmonary disease, CRT-D cardiac resynchronization therapy defibrillator, ICD implantable cardioverter-defibrillator, LVEF left ventricular ejection fraction, n.a. not applicable, NYHA New York Heart Association, SCA sudden cardiac arrest, SD standard deviation, VF ventricular fibrillation, VT ventricular tachycardia.
